# Case series of patients treated with Transcranial Magnetic Stimulation (TMS) in the psychiatry department of the Ciudad Real General University Hospital during the period 2024-2025

**DOI:** 10.1192/j.eurpsy.2026.10713

**Published:** 2026-07-31

**Authors:** D. E. Gerardino Salomon, C. Rodriguez-Gomez

**Affiliations:** Psiquiatria, Hospital General Universitario de Ciudad Real, Ciudad Real, Spain

## Abstract

**Introduction:**

This abstract presents a comprehensive overview of a case series examining the clinical application and outcomes of Transcranial Magnetic Stimulation (TMS) as a therapeutic intervention for patients with treatment-resistant depression (TRD), obsessive-compulsive disorder (OCD), and eating desorders (ED). The study, conducted at the Psychiatry Department of the Ciudad Real University General Hospital from 2024 to 2025.

**Objectives:**

The primary objective of this case series was to evaluate the clinical application and outcomes of TMS in a cohort of patients with TRD, OCD, and ED who had not responded to traditional pharmacological treatments.

**Methods:**

The methodology involved a retrospective analysis of patient data from individuals who underwent TMS treatment during the specified period. A total of 44 patients were included in the series, with a gender distribution showing a higher proportion of females. The age range of the patients was from 26 to 66 years, with the highest concentration of cases in the 40-50 age bracket. Protocols for depression, OCD and ED were distinctly applied, with specific parameters for frequency, pulses per train, number of trains, and inter-train intervals tailored to each condition, highlighting the personalized nature of the treatment. Patient outcomes were assessed through a validated questionary administered upon completion of the sessions. The results were categorized into three groups: remission, partial improvement, and no improvement.

**Results:**

The findings were promising, with a significant proportion of patients achieving full remission. Specifically, 58.2% of the patients experienced remission, 31.6% showed partial improvement, and only 10.2% reported no improvement. These results are particularly notable given that the cohort consisted of patients who had not responded to previous pharmacological interventions, underscoring TMS’s potential as a powerful alternative or adjunct therapy. The data on the average number of sessions per outcome category revealed a trend toward better results with a higher number of sessions, with high improvement correlating with a higher average session count compared to partial or no improvement. This suggests a dose-response relationship.

**Conclusions:**

Furthermore, the study confirmed that TMS is a well-tolerated technique with few side effects, reinforcing its status as a safe and non-invasive treatment modality. The consistency of these findings with those of previous, larger-scale international studies demonstrates the reproducibility and generalizability of TMS’s therapeutic benefits. However, as a case series, the study is limited by its small sample size. This limitation prevents rigorous statistical analysis and the drawing of definitive conclusions.

**Disclosure of Interest:**

None Declared

